# Thermal, Chemical and pH Induced Unfolding of Turmeric Root Lectin: Modes of Denaturation

**DOI:** 10.1371/journal.pone.0103579

**Published:** 2014-08-20

**Authors:** Himadri Biswas, Rajagopal Chattopadhyaya

**Affiliations:** Department of Biochemistry, Bose Institute, Calcutta, India; Russian Academy of Sciences, Institute for Biological Instrumentation, Russian Federation

## Abstract

*Curcuma longa* rhizome lectin, of non-seed origin having antifungal, antibacterial and α-glucosidase inhibitory activities, forms a homodimer with high thermal stability as well as acid tolerance. Size exclusion chromatography and dynamic light scattering show it to be a dimer at pH 7, but it converts to a monomer near pH 2. Circular dichroism spectra and fluorescence emission maxima are virtually indistinguishable from pH 7 to 2, indicating secondary and tertiary structures remain the same in dimer and monomer within experimental error. The tryptophan environment as probed by acrylamide quenching data yielded very similar data at pH 2 and pH 7, implying very similar folding for monomer and dimer. Differential scanning calorimetry shows a transition at 350.3 K for dimer and at 327.0 K for monomer. Thermal unfolding and chemical unfolding induced by guanidinium chloride for dimer are both reversible and can be described by two-state models. The temperatures and the denaturant concentrations at which one-half of the protein molecules are unfolded, are protein concentration-dependent for dimer but protein concentration-independent for monomer. The free energy of unfolding at 298 K was found to be 5.23 Kcal mol^−1^ and 14.90 Kcal mol^−1^ for the monomer and dimer respectively. The value of change in excess heat capacity upon protein denaturation (ΔC_p_) is 3.42 Kcal mol^−1^ K^−1^ for dimer. The small ΔC_p_ for unfolding of CLA reflects a buried hydrophobic core in the folded dimeric protein. These unfolding experiments, temperature dependent circular dichroism and dynamic light scattering for the dimer at pH 7 indicate its higher stability than for the monomer at pH 2. This difference in stability of dimeric and monomeric forms highlights the contribution of inter-subunit interactions in the former.

## Introduction

Lectins are a special group of sugar-binding proteins of non immune origin able to agglutinate cells and/or precipitate glucoconjugates. They have been identified in nature from microorganisms to animals and function as cognate receptors to various cell surface glycoproteins, resulting in several important cellular mediated events ranging from mitogenic processes to plant defense mechanisms [1], [2]. In recent years, plant lectins have become of increasing interest, mainly due to the discovery of their potent biological activities, and different lectins have been purified and characterized in some detail with respect to their biochemical properties and carbohydrate binding specificities [3], [4]. In addition the N-acetyl-d-glucosamine (GlcNAc) and mannose specific lectins generally manifest potent insulin like activities [5]. It has also been reported that the anti-insect activity as well as binding and phagocytosis of microorganisms in animals has generally been observed in the case of GlcNAc and mannose specific lectins [6], [7], [8], [9]. Consequently, to aid in the understanding of their function and in designing mimics, there is also an increasing interest in the molecular structure of plant lectins in relation to their functions [10].

The vast majority of all currently known plant lectins can be classified into seven families of evolutionary and structurally related proteins [11]. The lectin families are distinguished namely (i) the legume lectins [12], (ii) the chitin-binding proteins containing hevein domain(s) [13], (iii) the type 2 ribosome-inactivating proteins [14], (iv) the monocot mannose-binding lectins [15], (v) the *Cucurbitaceae* phloem lectins, (vi) the jacalin family and (vii) the *Amaranthaceae* lectins. The first lectin structures to be determined derived from two phylogenetically conserved families– the *Leguminoseae* [12] and the *Gramineae* [13]. The legume lectins display diverse sugar-binding specificities [16] and differences in their quaternary structures; however, they share a common polypeptide fold known as the 13-sandwich (or jelly roll) motif, consisting of three sets of antiparallel β-sheets connected by loops of diverse size [17]. The *Gramineae* family lectins belonging to a super family of chitin-binding proteins, have also been studied extensively [13]. These dimeric proteins have a most distinctive subunit fold that consists of four independently folded and helically assembled 43-residue segments. These lectins have evolved by gene duplication of an ancestral disulfide-rich domain [18] lacking significant amounts of regular secondary structure [19]. The first three monocot lectin structures determined were of *Galanthus nivalis*, family (iv) of β-prism II fold [20]; jacalin, of family (vi) of β-prism I fold [21]; and *Amaranth caudatus* seeds of family (vii) of β-trefoil fold [22] respectively, their folds stabilized by conserved hydrophobic side chains which line the core of the β-barrel. All three are relatively small proteins of 12–16 kD per subunit or domain, and possess subunit structures that display pseudo threefold symmetry.

*Curcuma longa*, commonly called turmeric, is widely used as food, flavoring, coloring, spice agent, a traditional herb and belongs to the *Zingiberaceae* family. A purified dimeric *Curcuma longa* rhizome lectin (CLA) is a glycoprotein with about 5.2% covalently bound sugar. The lectin possessing antifungal, antibacterial and α-glucosidase inhibitory activities also showed apparent thermal stability for incubating temperatures below 333 K, as well as acid tolerance till pH 2, by measuring agglutination activity [23]. Despite this study [23] on the turmeric lectin, the molecular basis for its stability and assembly remains unknown. The relationship between protein stability and oligomerization of turmeric lectin (*Zingiberaceae* family) has been highlighted for the first time in our study.

Lectin unfolding was studied by differential scanning calorimetry (DSC) at different pH values, protein concentrations and scan rates. The oligomeric state of the lectin at different pH values were observed by size-exclusion chromatography and dynamic light scattering (DLS). The effect of pH on secondary and tertiary structures of the lectin was investigated by circular dichroism (CD) spectroscopy. Chemical unfolding of turmeric lectin by GdnCl was monitored by changes in the fluorescence characteristics and CD spectra of the lectin at different temperatures.

## Materials and Methods

### Materials

Ultrapure guanidinium chloride (GdnCl) and ammonium sulfate were purchased from Calbiochem. Concanavalin A sepharose, HiTrap Q HP column, Superdex 75 pg column and gel filtration molecular mass standards were purchased from GE Healthcare Biosciences Ltd. Biogel P-100 was purchased from BioRad. A protein molecular mass marker set was purchased from Fermentas.

### Protein purification

Fresh rhizomes of *C. longa* were purchased from a local market, were grounded after peeling and cut into small pieces adding 20 mM Tris-HCl, 1 mM CaCl_2_, 1 mM MnCl_2_, 150 mM NaCl (pH 7.4) 1∶5 (w/v) using a homogenizer. The extraction and purification procedure using Concanavalin A sepharose column was otherwise similar to the published procedure [23]. Final elution of the protein was carried out using 0.5 M methyl-α-D-glucopyranoside in the same buffer. Fractions eluted from Concanavalin A sepharose column having hemagglutination activity were pooled and dialyzed against 20 mM Tris-HCl (pH7.2). For measuring this activity, rabbit erythrocytes were washed 5 times with PBS (phosphate buffer saline, 10 mM potassium phosphate buffer pH 7.2 containing 150 mM NaCl). A 2% (v/v) suspension of the erythrocytes in the same buffer was prepared and assays performed [24]. The dialyzed sample was then loaded on a Hitrap Q HP column coupled to the AKTA prime plus (GE Healthcare) system, pre equilibrated with 20 mM Tris-HCl (pH 7.2). The protein was then eluted using a linear gradient of 0 to 0.35 M NaCl with a flow rate of 1 ml min^−1^. The active fractions were collected and further dialyzed against 50 mM PBS. The dialyzed protein was then concentrated using Centriprep-10 (Millipore) and loaded on Superdex 75 pg column pre equilibrated with 0.2 M methyl-α-D-glucopyranoside in 50 mM PBS at a flow rate of 1 ml min^−1^. The active fractions were collected and analyzed by SDS-PAGE.

### Amino acid analysis and protein concentration determination

The amino acid compositions of experimental samples were determined using the HPLC-Pico-Tag method. Protein concentrations were determined spectrophotometrically using an extinction coefficient of 13,980 M^−1^ cm^−1^ at 280 nm.

### pH treatment of CLA

To study the effect of pH on the secondary structure of CLA, the following buffers were used having 150 mM NaCl: 10 mM Glycine-HCl (pH 1–3), 10 mM Na-acetate (pH 4–5), 10 mM Na-phosphate (pH 6–7). Protein was incubated for 5 h at room temperature at different pH values before recording various spectroscopic measurements, size exclusion chromatography, dynamic light scattering or differential scanning calorimetry as described below.

### Size-exclusion chromatography

Size-exclusion chromatography was performed to determine the oligomeric state of CLA at different pH values using a Biogel p-100 column. The column volume 50 ml was determined by vitamin B_12_ and void volume (V_0_) of 16 ml by blue dextran. CLA was incubated in various buffers as already described. All standards (Conalbumin, 75 kD; Ovalbumin, 43 kD; Chymotripsinogen, 25 kD; Ribonuclease A, 13.7 kD) were run at pH 7. The standard curve was drawn by plotting the ratio of elution volume to void volume (V_e_/V_0_) against logarithm of relative molecular mass.

### Dynamic light scattering

DLS measurements were carried out at 830 nm with a Malvern Nano instrument equipped with a temperature-controlled micro sampler, using a protein concentration of 37.5 µM. Before measurement, all the solutions were spun at 12,000 rpm for 15 min and filtered through 0.22 µm filter. Protein was incubated at different pH values of 2 to 7. Data were analysed by using Dynamics 6.10.0.10 software at optimized resolution. The mean hydrodynamic radius (R_h_) and polydispersity (Pd) were estimated on the basis of an autocorrelation analysis of scattered light intensity based on the translational diffusion coefficient, by Stokes–Einstein equation:
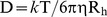
(1)Where R_h_ is the hydrodynamic radius, *k* is the Boltzmann's constant, T is the absolute temperature, η is the viscosity of solvent and D is the translational diffusion coefficient. Hydrodynamic radius (R_h_) as a function of temperature was determined by dynamic light scattering for pH 7 and pH 2.

### Circular Dichroism Spectroscopy

Circular dichroism measurements were carried out at 298 K temperature with a J-800 JASCO spectrometer using a quartz cell of 1 mm path length. For far-UV analysis the spectra were recorded in the range of wavelengths 260–210 nm at a scan speed of 100 nm min^−1^ with a response time of 1s and slit width 1 nm. The lectin concentration used was 10 µM for all the samples. Each spectrum, including that measured for buffer alone for the purpose of baseline determination, was obtained as an average of 3 scanned ones. The spectra presented are after baseline subtraction.

Temperature-induced denaturation of the CLA secondary structures were monitored by following the loss of ellipticity at 227 nm, using CD spectroscopy. The temperature at midpoint of transition (T_m_) was determined by plotting 0.5 unit of fraction native. The effect of temperature on the secondary structure of CLA was studied at pH 7 and 2 by increasing the temperature of the protein samples at the rate of 1 K min^−1^ in the temperature range of 298–368 K with the interval of 2 K. All data were corrected for respective buffer base line. Reversibility of protein unfolding was checked by return of the complete spectrum upon thermal unfolding followed by subsequent cooling to ambient temperature.

To study the tertiary structure of CLA at pH 7 and 2, the near-UV spectra were recorded at a protein concentration of 30 µM in the range of 250–300 nm using a cuvette of path length 5 mm.

### Steady state Fluorescence Spectroscopy

Intrinsic fluorescence of the proteins were measured using a Hitachi F3010 spectrofluorimeter at a scanning speed of 240 nm per min in 1 cm path length quartz cuvette having slit widths of 2.5 and 5 nm for excitation and emission, respectively. The lectin solution (5 µM) was excited at 295 nm and emission spectra were recorded between 305–400 nm. To observe the effect of pH on fluorescence maximum, CLA was incubated at different pH values from 1 to 7 in the respective buffers for 5 h. Different spectra were analyzed by subtracting buffer spectra from protein spectra yielding the corrected spectra shown.

Fluorescence quenching measurements were carried out at pH 7 and pH 2 by adding small aliquots of acrylamide (5 M) by titrating to the protein (5 µM) samples. Protein samples were excited at 295 nm and emission spectra were recorded at 305–400 nm. The fluorescence intensities obtained were corrected for dilution. Quenching data were analyzed using the Sterne-Volmer equation:
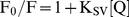
(2)where F_0_ and F are the emission intensities in the absence and presence of the quencher, [Q] is the molar concentration of the quencher and K_SV_ is the Sterne-Volmer quenching constant.

For the ANS binding experiments, a stock of ANS was prepared in distilled water and protein to ANS molar ratio was maintained as 1 : 10. The excitation wavelength was set at 375 nm and emission spectrum was recorded at 430 to 550 nm.

### GdnCl induced unfolding of CLA and its analysis

Isothermal GdnCl-iduced unfolding studies of native CLA were performed in the temperature range 288 K to 333 K and monitored using fluorescence spectroscopy and far-UV circular dichroism. GdnCl stock solutions, 8 M, were freshly prepared either in 10 mM Na-Phosphate (pH 7) or 10 mM glycine-HCl (pH 2) having 150 mM NaCl. CLA samples were brought to 0–7 M (for pH 7) and 0–6 M (for pH 2) in GdnCl with these buffers. Fluorescence and CD measurements were done under same experimental conditions as stated earlier, using 5 µM protein concentrations. For concentration dependent study 2 µM and 10 µM protein sample were used at the desire temperature.

Assuming that unfolding of CLA follows a two-state model, the fraction of unfolded protein molecules (f_u_) was calculated from the following equation:
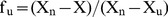
(3)Where X, X_n_, and X_u_ represent the observed spectroscopic signal of the protein at any denaturant concentration, the spectroscopic signal of the protein in the completely folded state, and the spectroscopic signal of the protein in the completely unfolded state, respectively.

C_m_ (denaturant concentration at the midpoint of unfolding transition) values of the two-state pathways was determined by nonlinear fitting of the unfolding data to eq 4 using GraphPad Prism (GraphPad Software Inc.) as described [25]

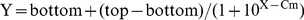
(4)Where X and Y represents the concentration of unfolding agent and the fraction of unfolded protein molecules respectively.

The change in free energy of unfolding in water (ΔG°) is obtained by linear extrapolation model [26]. The relationship between the denaturant and ΔG° is approximated by the following equation:
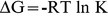
(5)and
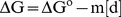
(6)Where m is the experimental measure dependence of ΔG; ΔG° is the free energy change in absence of denaturant, its concentration denoted by [d]. R is the gas constant (1.987 cal K^−1^ mol^−1^) and T is 298 K.

After obtaining ΔG° at each temperature, the values were fitted to the following equation to obtain ΔH_g_, ΔC_p_ and T_g_ [27]

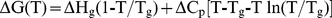
(7)


### Differential scanning calorimetry

DSC experiments were performed on a Microcal VP-DSC micro calorimeter equipped with two fixed cells, a reference cell and a sample cell. DSC experiments were carried out as a function of pH, protein concentration and scan rate. For DSC experiments, 40 µM protein solution was used, varying the pH from 2 to 7 using buffers described. Prior to all the measurements the buffers and protein solutions were degassed and protein was dialyzed against respective buffers overnight. The DSC scans were run between 298 K and 373 K. Respective reference scan was run under identical DSC setup conditions and subtracted from each sample scan.

The van't Hoff enthalpy (ΔH_v)_ associated with the DSC thermograms were determined by the two-state fit of the thermogram using the Origin software supplied by MicroCal assuming N_2_↔2U type transition, where N represents the native monomer and U the unfolded monomer, while the calorimetric enthalpy (ΔH_c_) was determined by the area under the transition. T_m_ and T_p_ are the temperatures of half of the transition peak area and the temperature at which the transition peak is at its maximum, respectively.

The dependence of T_p_ upon protein concentration for an oligomeric protein undergoing dissociation can be expressed by following equation [28]

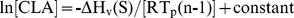
(8)Where n is the number of subunits per oligomer, T_p_ is the temperature corresponding to the transition peak maximum, [CLA] concentration of the protein and ΔH_v_(S) is the van't Hoff enthalpy obtained by the slope of ln[CLA] vs 1/T_p_.

## Results

### Protein purification

**Figure 1:**
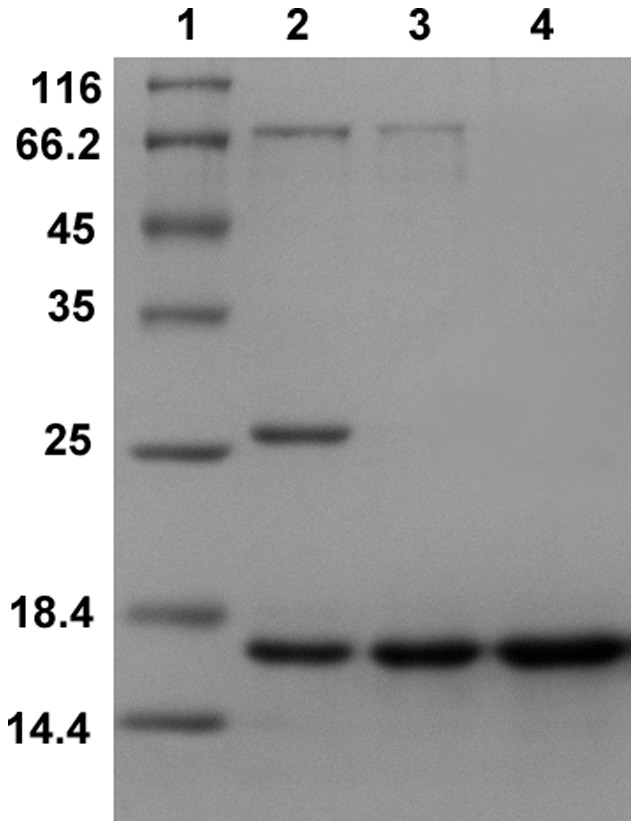
Purification of CLA using different chromatographic techniques. Lanes 1–4 represent molecular mass markers of indicated masses in kilo Daltons, purified protein after Con A sepharose, Q-sepharose and Superdex 75 pg columns respectively. The purified glycosylated protein had a molecular mass of 16 kD by comparison with lane 1.

Following extraction from fresh rhizomes, ammonium sulfate precipitation, the precipitate containing the lectin was purified as described and judged to be extremely pure (>98%) (Fig. 1).

### Amino acid analysis and protein concentration determination

The results of our amino acid analysis are summarized in Table 1. The protein seems to be rich in Asx, Glx, Gly, Ile and Leu, but no Cys residue was found. The number of Trp residues cannot be found by the pico-Tag method as it gets destroyed by the digestion protocol using high temperature. Therefore it was determined by an old spectroscopic method by measuring protein spectra in 0.1 N NaOH at 294.4 nm and 280 nm [29]. The calculated molecular mass of deglycosilated CLA is 15,021.28 Daltons, whereas the molecular mass determined by MALDI-TOF mass spectrometry of deglycosilated protein gave a molecular mass of 14,947.0 Daltons (data not shown). The extinction coefficient was calculated to be 13,980 M^−1^ cm^−1^ at 280 nm using the number of Trp and Tyr residues shown in Table 1.

**Table 1:** Amino acid composition of *Curcuma longa* lectin.

Amino Acids	Fraction of residues	Number of residues	Nearest integer
Asx	0.1743	23.71	24
Glx	0.1031	14.02	14
Ser	0.0562	07.00	07
Gly	0.1350	18.36	18
His	0.0201	02.73	03
Arg	0.0194	02.63	03
Thr	0.0615	08.36	08
Ala	0.0332	04.51	05
Pro	0.0635	08.64	09
Tyr	0.0159	02.16	02
Val	0.0150	02.05	02
Met	0.0041	00.55	01
Cys	0.0000	00.00	00
Ile	0.1076	14.63	15
Leu	0.1235	16.80	17
Phe	0.0272	03.70	04
Lys	0.0404	05.49	05
Trp	n.d.	n.d.	02

* – the method of Trp determination was an old spectroscopic method of Goodwin and Morton [29].

### Size-exclusion chromatography

**Figure 2:**
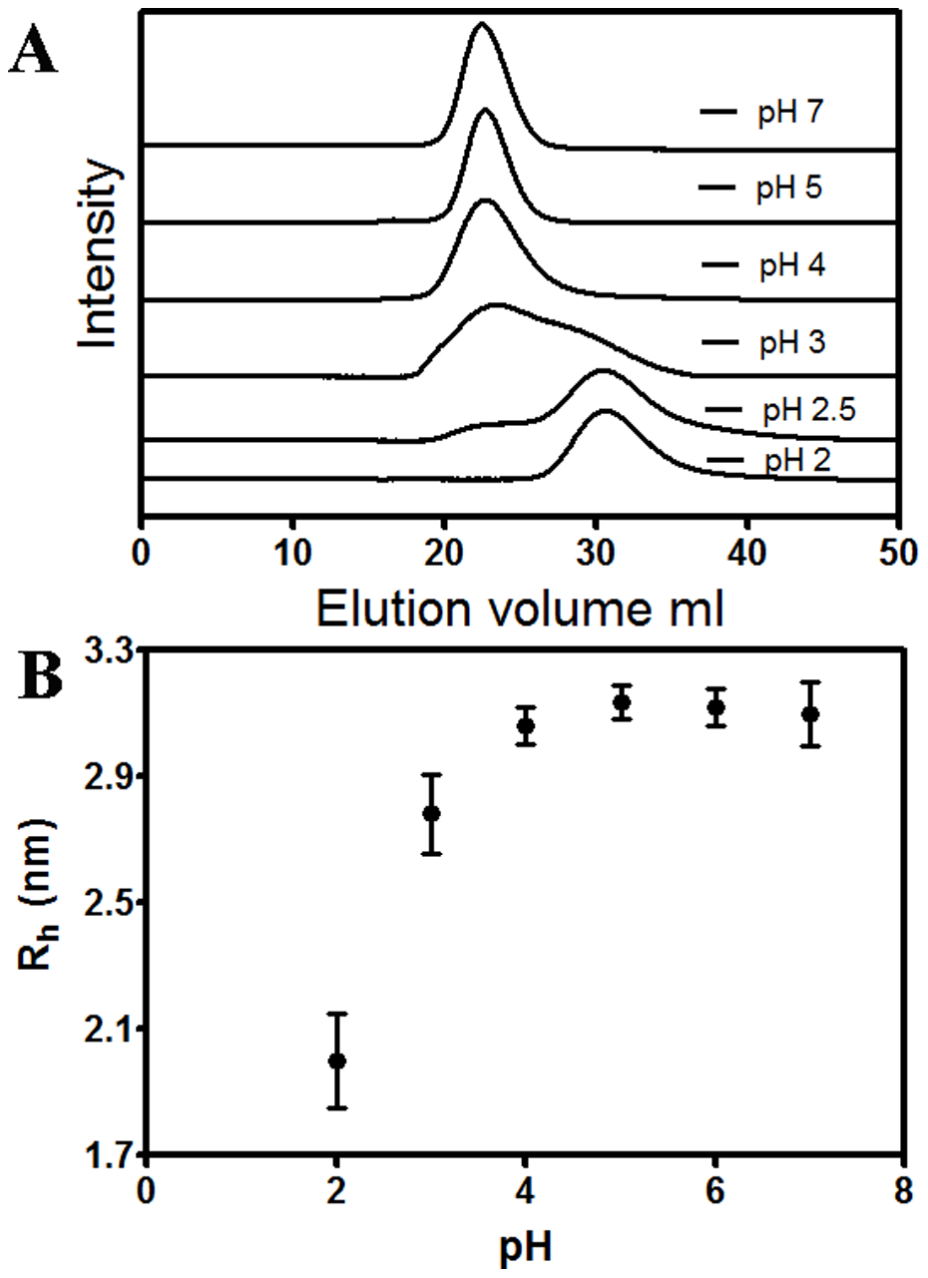
Size-exclusion chromatography and dynamic light scattering studies of CLA at different pH values. A) Gel filtration profiles for pH 7, 5, 4, 3, 2.5 and 2 from top to bottom, in that order. It is apparent that in the bottom profile at pH 2, the peak maximum elutes around 31 ml representing a monomeric species, while the three uppermost profiles at pH 7, 5, 4 represent a dimeric species of CLA and both species are present at the intermediate pH values of 3 and 2.5. The maximum peak height was approximately 0.020 absorbance units at 280 nm for each of the profiles. B) pH dependent dynamic light scattering at protein concentration of 37.5 µM. The hydrodynamic radius of dimer and monomer are 3.1±0.2 nm and 2.0±0.2 nm, respectively.

Gel-filtration studies using a Bio-rad P-100 column were performed in the pH range 2–7 (Fig. 2A). There was an appreciable change in the position of the CLA peak at pH 2 relative to higher pH measurements, and elution volume increased progressively from 22.44 ml to 30.63 ml in the range of pH 7-2. From a standard curve, the molecular mass was calculated to be 16 kD for that peak eluting ∼31 ml. It is noticed that for the experiments at pH 4, 5 and 7, the maxima of the peak is ∼22 ml (Fig. 2A). At intermediate pH values 3 and 2.5, the presence of two peaks eluting ∼31 ml and ∼22 ml are noticed. After lowering pH to 2, *only* the 16 kD peak eluting ∼31 ml was observed. This implies, upon lowering pH from 7 to 2, CLA converts from dimer to monomer.

### Dynamic light scattering

**Figure 3:**
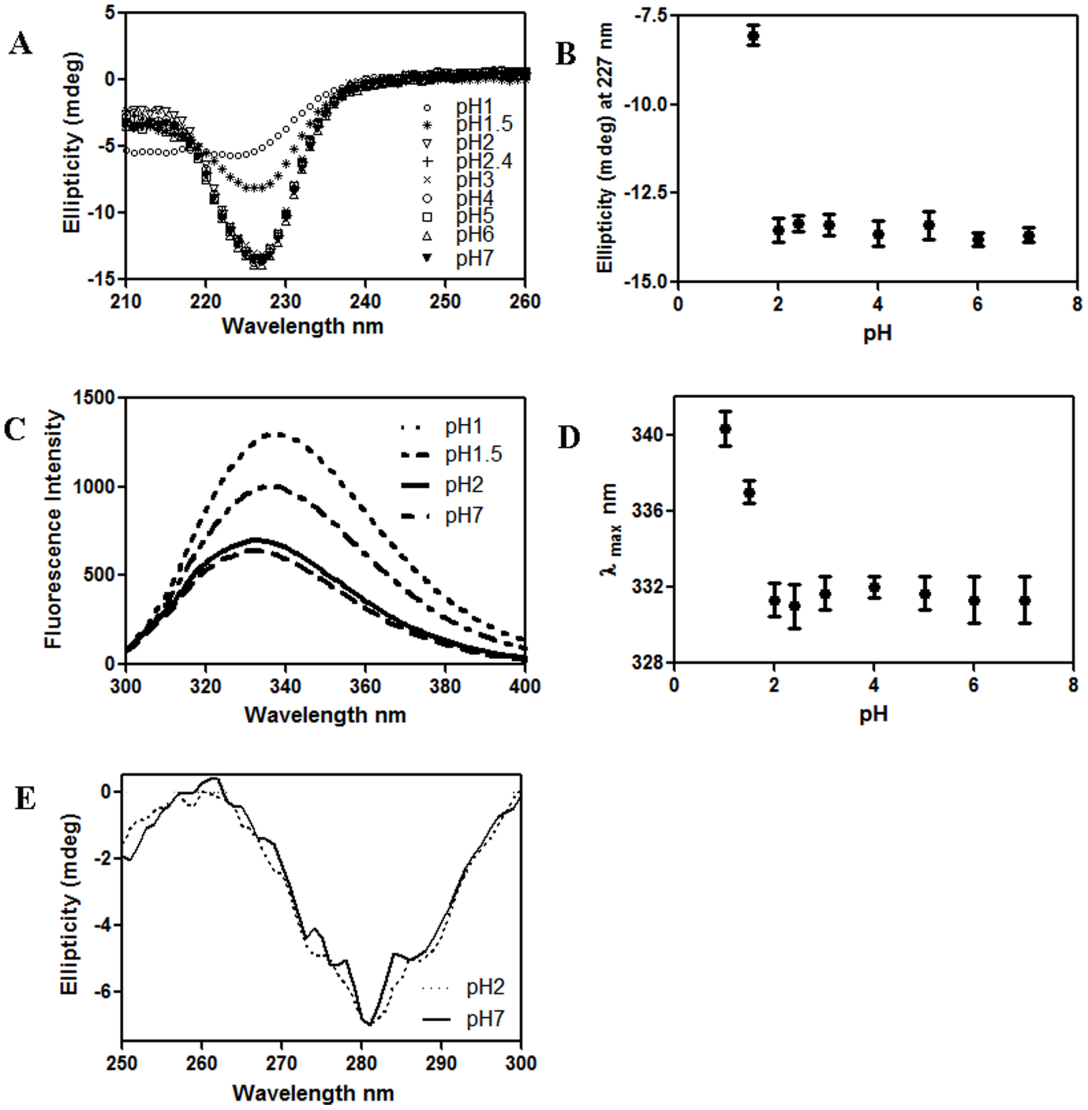
Temperature dependence of hydrodynamic radius, R_h_. The variation of R_h_ as a function of temperature at pH 2 (□) and pH 7 (○) is shown. DLS was carried out at a scan rate of 1 K min^−1^ using a protein concentration of 37.5 µM.

Dimerization in CLA can be detected by DLS measurements where an increase of hydrodynamic radii (R_h_) as well as elevation in percent polydispersity takes place. Fig. 2B shows the plot of hydrodynamic radius against pH for CLA. Under these conditions, polydispersity varies from 10% to 22% from pH 2 to pH 7. It was found that CLA has a hydrodynamic radius of 3.1±0.2 nm in the pH range 4 to 7. The size gradually starts changing below pH 4; finally, at pH 2 it attains a size of 2.0±0.2 nm. The higher polydispersity (22%) and R_h_ (3.1±0.2 nm) in neutral pH conditions are more favorable for oligomerization. On the other hand, the lower polydispersity (10%) and R_h_ (2.0±0.2 nm) in pH 2 apparently suggest monomeric units. The CLA size of 3.1 nm in the pH range 4 to 7 indicates that the species is normally a dimer. At pH 3, there is a drop in size to 2.8 nm. Temperature-dependent DLS (Fig. 3) was performed on both forms of the protein (i.e., pH 7 and 2) from 298 to 368 K. For pH 7, in the temperature range 298–333 K, the average hydrodynamic radius is about 3.1 nm; at 368 K it reaches 3.8 nm. At pH 2, the average hydrodynamic radius is about 2.0 nm in the range 298–313 K, beyond 313 K, R_h_ increases indicating unfolding of monomer.

### Circular Dichroism Spectroscopy

**Figure 4:**
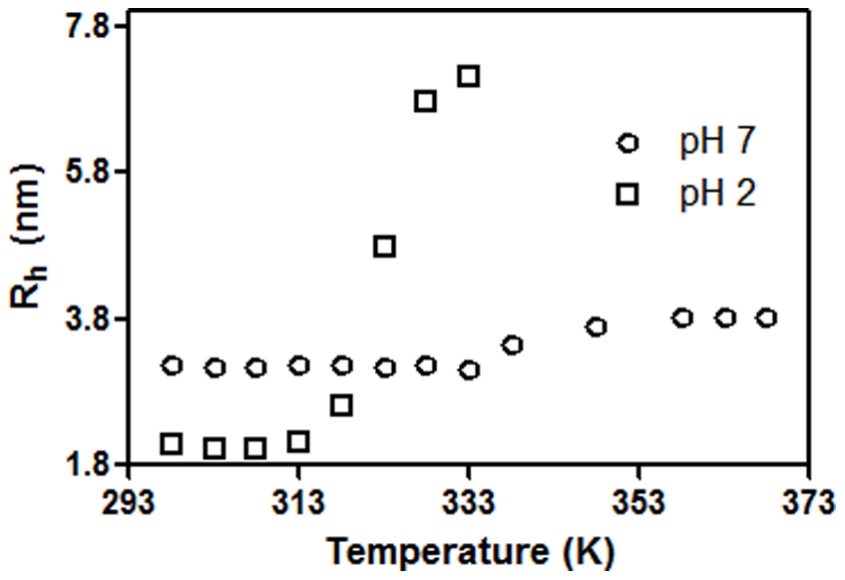
Characterization of CLA at different pH values by spectroscopy. A) Far UV CD spectra (specific ellipticity) of 10 µM CLA shown at different pH values as mentioned in the diagram. These data suggest that the secondary structure stays unchanged in the pH range 2–7, but is gradually lost below pH 2. At pH 1, roughly 40% of the secondary structure remains compared to pH 2. B) Ellipticity at 227 nm as a function of pH. C) Fluorescence intensity of CLA as a function of emission wavelengths at pH 1, 1.5, 2 and 7. D) Emission maximum (λ_max_) plotted as function of pH. E) Near UV spectra, hence tertiary structures, of 30 µM CLA at pH 2 (monomer) and pH 7 (dimer) are virtually identical.

An earlier report suggested that its activity peaked in the pH 2–7 range [23]. There was almost no change in secondary structure observed by specific ellipticity in the range pH 2 to 7, but as pH drops below 2, CD signals start dropping in magnitude and at pH 1, it goes down to about 40% of its maximum value at 227 nm (Fig. 4A). Secondary structure prediction by CDNN program [30] using the spectrum at pH 7 indicates CLA is a β protein containing frequent turns with 11% α-helix, 40% β-sheet and 22% turns. The spectrum at pH 2 yielded identical results by CDNN. The near UV spectra at pH 2 and pH 7 are virtually identical, with minima around 282 nm (Fig. 4E).

**Figure 5:**
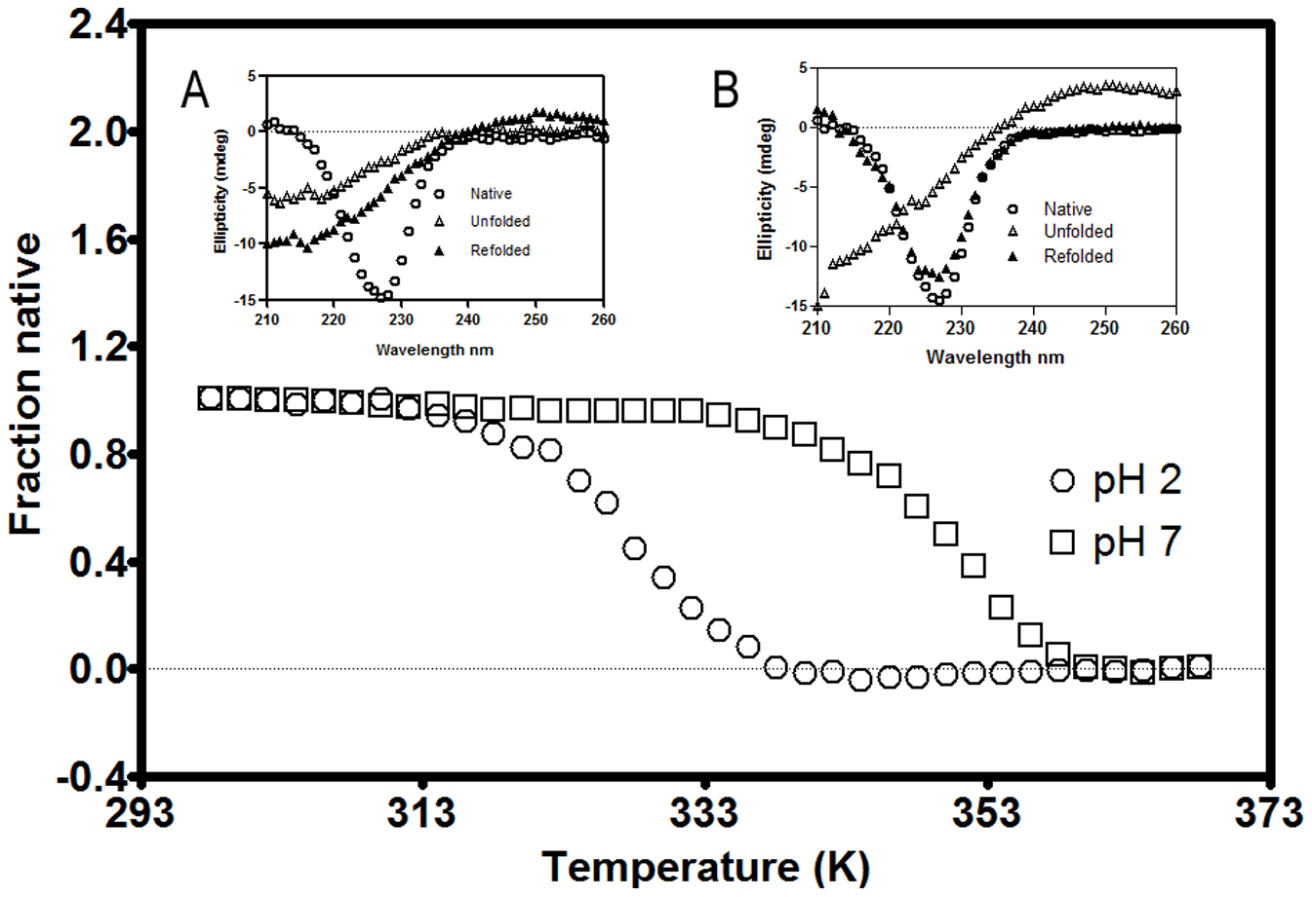
Thermal denaturation of dimeric and monomeric CLA. Thermal denaturation profiles for CLA at pH 2 (□) and pH 7 (○) as monitored by the change in ellipticity values at 227 nm upon temperature increase. Insets A and B represent native (○), unfolded (Δ) and refolded (▴) CD spectrum at pH 2 and pH 7 respectively.

The temperature dependent value of ellipticity at 227 nm was plotted for CLA at pH 7 and pH 2 (Fig. 5). The melting temperature was determined to be 327.5 K for pH 2 and 351.2 K for pH 7. Hence the thermal stability of CLA is higher at pH 7 relative to that at pH 2. To check the reversibility of thermally unfolded CLA at pH 7and pH 2, protein samples were brought to 298 K and rescanned. At pH 7 it was observed to be approximately 90% reversible (Fig. 5 inset B) but at pH 2 the transition was observed to be irreversible (Fig. 5 inset A).

### Steady State Fluorescence Spectroscopy

**Figure 6:**
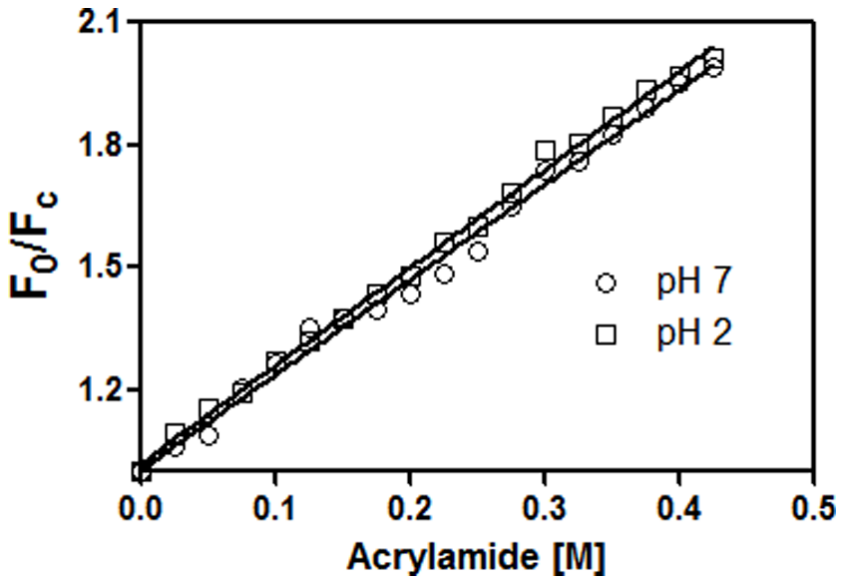
Exposure of tryptophan was monitored by acrylamide quenching. Stern Volmer plots for quenching CLA intrinsic fluorescence at different pH with acrylamide. Quenching measurements were performed at pH 2.0 (□) and pH 7.0 (_○_).

The intensity and the maxima of the emission spectra are used to characterize protein molecular structures as tryptophan residues are highly sensitive to their surrounding environment. The emission λ_max_ of CLA for pH 2 to 7 is seen at 330±2 nm; therefore the tryptophan environments of CLA do not change in this pH range (Fig. 4C). Moreover, the λ_max_ of the unfolded protein is close to that of free tryptophan and shows a substantial increase in fluorescence intensity. The unaltered tryptophan environment is also observed by fluorescence quenching measurements, in which the neutral acrylamide quenched about 50% of the protein intrinsic fluorescence at 0.425 M concentration (data not shown). The quenching profiles with acrylamide at pH 2 and pH 7 give similar results (Fig. 6 & Table 2). In the native state at pH 7, CLA does not bind to ANS, whereas at pH 2 substantial binding of ANS was observed (data not shown).

**Table 2:** Acrylamide quenching constants (K_sv_) of dimeric (pH 7), monomeric (pH 2) and denatured states (6 M GdnCl) of CLA.

Conditions	K_sv_ M^−1^	R^2^
pH 7	2.38	0.98
pH 2	2.40	0.98
6 M GdnCl	16.14	0.98

### GdnCl induced unfolding of CLA

**Figure 7:**
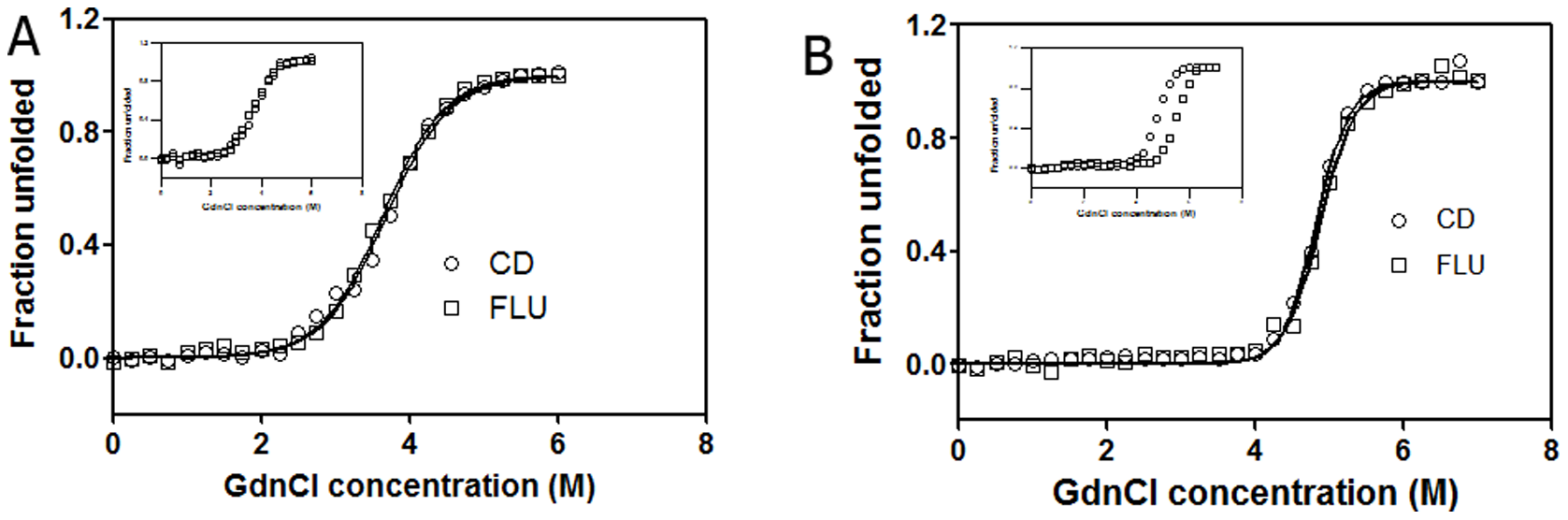
Chemical unfolding was monitored by CD and fluorescence. Fraction unfolded at 298 K temperature computed by a two-state model using equations (4) and (6) using CD (○) (specific ellipticity at 227 nm, 5 µM lectin) and Fluorescence (□) (emission intensity at 360 nm, 5 µM protein) for CLA as a function of the denaturant concentration at pH 2 (A) and pH 7 (B). (Insets) Concentration dependent GdnCl-induced unfolding monitored by change in fluorescence emission at 360 nm at pH 2 and pH 7 with 2 µM (○) and 10 µM (□) lectin respectively.

The denaturation profiles of dimeric and monomeric CLA are monitored by fluorescence and CD isothermal melts. The ellipticity at 227 nm was the most sensitive indicator as for different values of [GdnCl] it varied more than those at other wavelengths studied. The intrinsic fluorescence at 360 nm was used as a probe to monitor the change, as at this wavelength there was maximum difference between the intensity of the native and denatured species. The curve fitted well to a two-state model described by Eq. 4 and Eq. 6 (r-square value = 0.99), by the observation that fluorescence and CD plots can be superimposed (Fig. 7).

**Figure 8:**
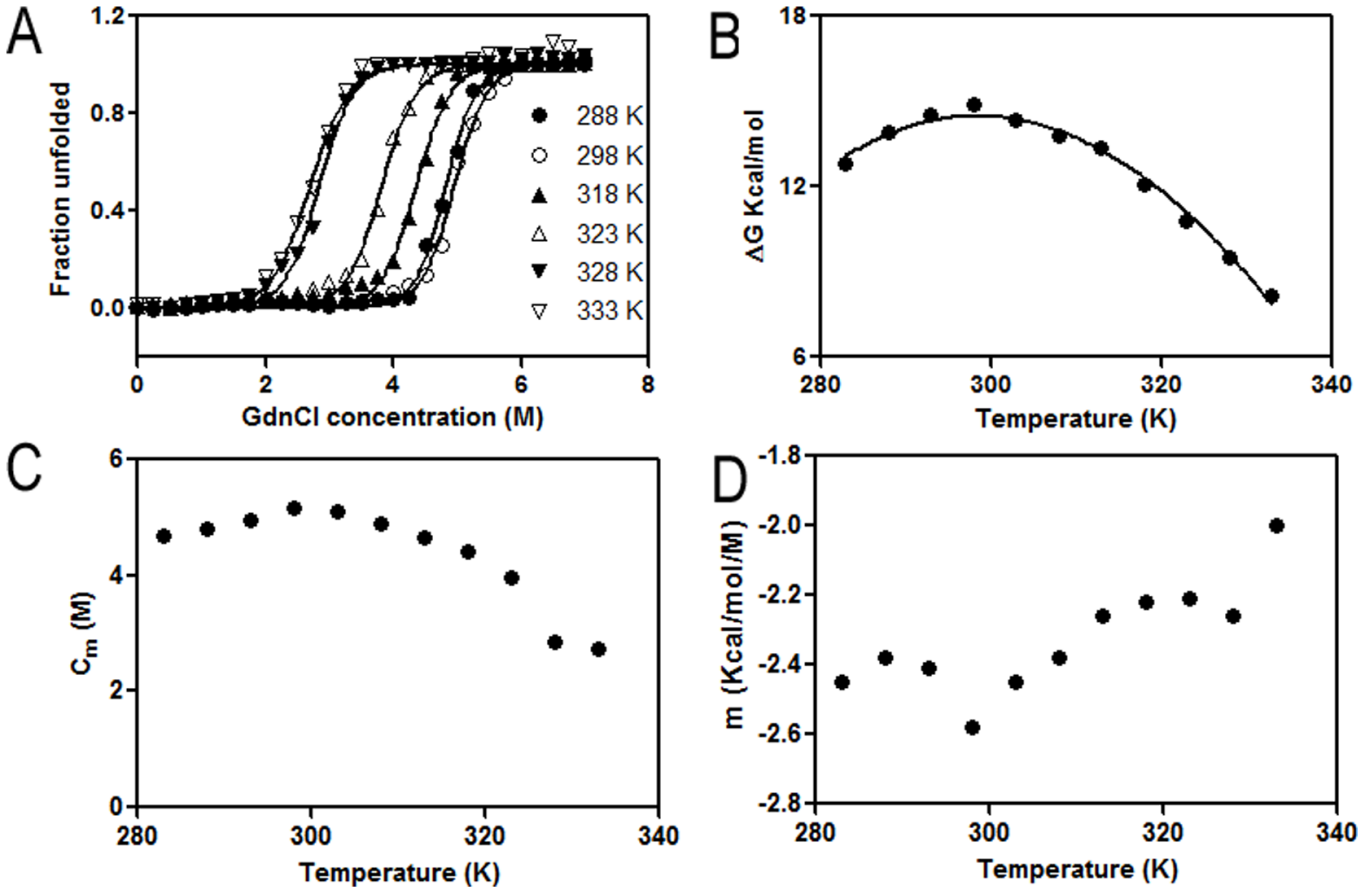
Unfolding free energy, C_m_ and m as a function of temperature. A) Overlay of GdnCl-induced isothermal melts done at different temperatures ranging from 283 to 333 K for pH 7, monitored by fluorescence at 360 nm. B) Thermodynamic stability of the dimer (○) at pH 7. Stability curves are derived by fitting the GdnCl-induced isothermal denaturation data to Eq. 7. C) C_m_ as a function of temperature for the GdnCl denaturation curves in the temperature range 288–333 K. D) m (obtained from fitting the GdnCl denaturation profiles to Eq. 6 as function of temperature.

The C_m_ of dimeric CLA increases at 298 K from 4.96±0.06 M at 2 µM protein to 5.38±0.02 M at 10 µM protein (data not shown), consistent with an N_2_↔2U denaturation transition. The C_m_ of monomeric CLA was found to be independent in the range of protein concentrations studied (2–10 µM), with an average value of 3.68±0.1 M (results not shown). Denaturation data at pH 2.0 were fitted to a N↔U transition. The mean values of ΔG°, C_m_ and m were 5.2±0.2 kcal mol^−1^, 3.68±0.1 M and −1.41±0.2 kcal mol^−1^ for the monomer, and 14.9±0.6 kcal mol^−1^, 5.18±0.2 M and −2.54±0.2 kcal mol^−1^ for the dimer respectively at 298 K (Table 3). From each of the isothermal melts, ΔG° of unfolding at each temperature for dimer (Fig. 8A, Table 4) was calculated and it can be clearly seen that the protein becomes less stable at temperatures above and below 298 K, particularly above 323 K (Fig. 8C, Table 4). The value of m, defined in Eq. 6, varies between −2.6 to −2.0 (Fig. 8D). The values of ΔH_g_, ΔC_p_ and T_g_ generated from the stability curve by fitting the values of ΔG° and T to Eq. 7 (Fig. 8B, Table 5).

**Table 3:** Thermodynamic parameters derive from GdnCl-induced unfolding at 298 K for the stability of CLA at pH 7 and pH 2.

Condition	Unfolding probe	C_m_ (M)	ΔG° (Kcal mol^−1^)
pH 7	FI at 360 nm	5.18	14.90±0.66
pH 7	CD at 227 nm	5.24	14.66±0.71
pH 2	FI at 360 nm	3.68	5.21±0.21
pH 2	CD at 227 nm	3.62	5.25±0.15

**Table 4:** Free-energy changes upon unfolding for pH 7 at different temperature obtained from GdnCl-induced isothermal denaturation melts.

T (K)	ΔG° (Kcal mol^−1^)	-m (Kcal mol^−1^ K^−1^)	C_m_ (M)
283	12.80±0.51	2.45±0.03	4.70
288	13.86±0.41	2.38±0.01	4.81
293	14.49±0.38	2.41±0.01	4.97
298	14.90±0.66	2.58±0.02	5.18
303	14.35±0.31	2.45±0.03	5.11
308	13.81±0.29	2.38±0.02	4.91
313	13.33±0.37	2.26±0.05	4.66
318	12.10±0.21	2.22±0.03	4.42
323	10.81±0.33	2.21±0.06	3.97
328	9.59±0.35	2.26±0.05	2.86
333	8.19±0.41	2.00±0.09	2.72

**Table 5:** Thermodynamic parameters of CLA dimer analysed on the basis of stability curves drawn by fitting data to Eq. 7.

ΔH_g_ (Kcal mol^−1^)	T_g_ (K)	ΔC_p_ (Kcal mol^−1^ K^−1^)
191.10±14.07	350.1±2.4	3.42±0.5

**Figure 9:**
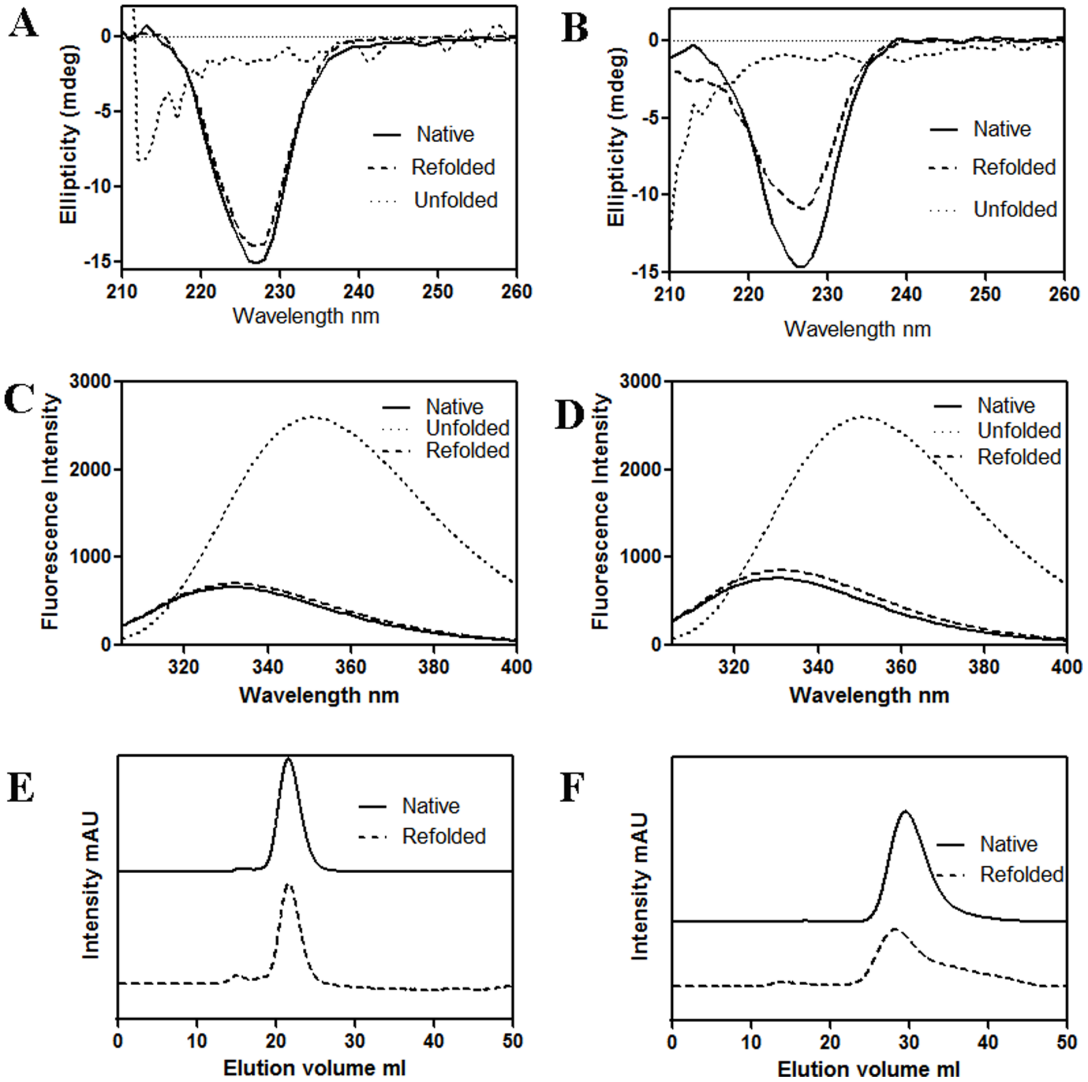
Refolding of CLA. At pH 2 and pH 7, the refolding of CLA was monitored by CD, fluorescence and gel filtration chromatography. A) CD, C) Fluorescence and E) Gel filtration of CLA at pH 7. B) CD, D) Fluorescence and F) Gel filtration at pH 2.

To check the reversibility of chemical unfolding of CLA at pH 7 and pH 2, protein samples were unfolded by GdnCl, then samples were diluted 10 fold. Fig. 9 shows the refolding at two pH values, monitored by CD, fluorescence and gel filtration, respectively. At pH 2 and pH 7, reversibility was observed approximately at 70% and 90% levels respectively. The λ_max_ of the unfolded protein is significantly red-shifted at 350 nm with respect to that in the folded protein (both for monomer and dimer) (Fig. 9C, 9D).

### Differential scanning calorimetry

**Figure 10:**
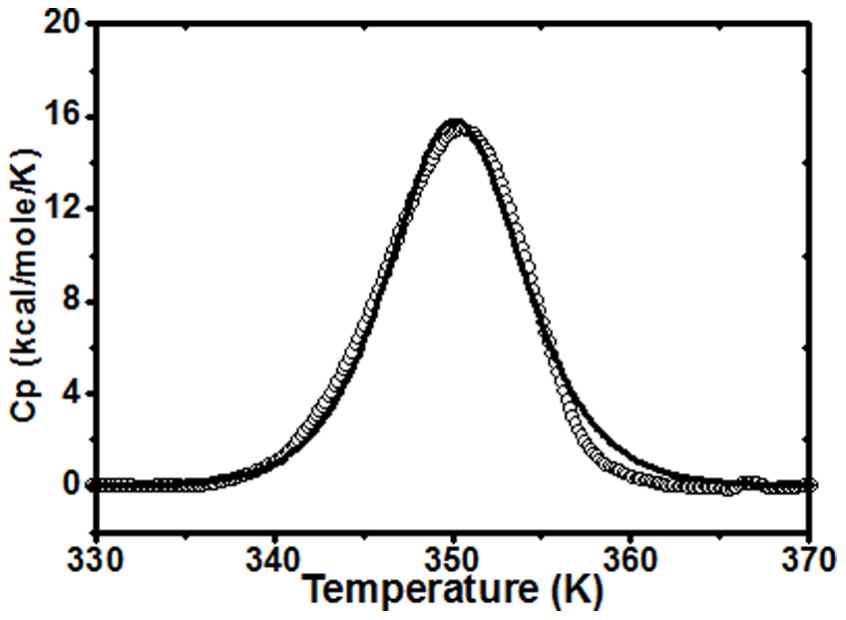
DSC scan of CLA. Excess heat capacity curves of 40 µM CLA at pH 7 was monitored by differential scanning calorimetric study at a scan rate of 60 K h^−1^. The solid line represents the best fit of the DSC data to a two state transition model.

**Figure 11:**
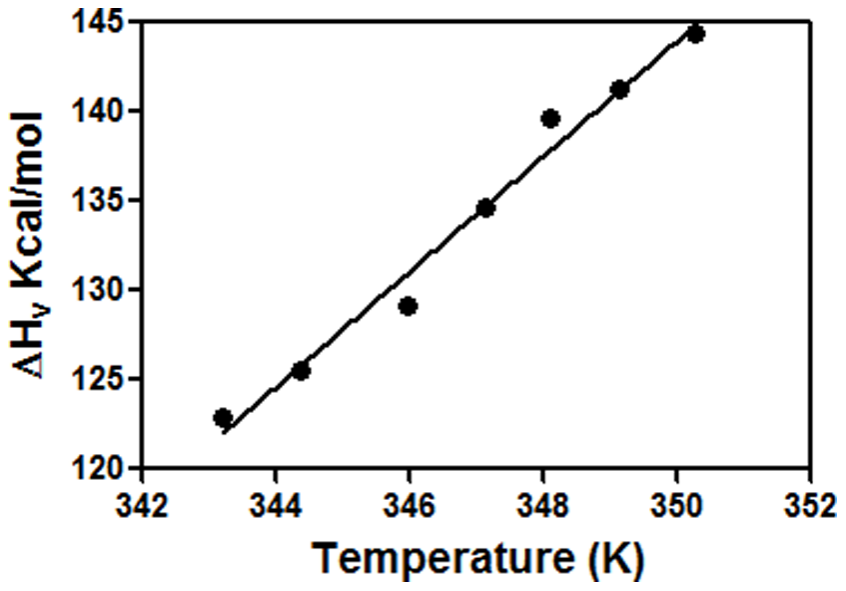
Slope of the plot of enthalpy obtained from individual DSC runs. ΔH_v_ vs. T_m_ yields the value of ΔC_p_ = 3.23 Kcal mol^−1^ K^−1^. The different pH points used for the study were 4, 4.5, 5, 5.5, 6, 6.5 and 7 (correlation coefficient = 0.98).

In this study, the thermal stability of *C. longa* rhizome lectin has been investigated under different conditions by using high-sensitivity DSC measurements. Effect of pH on the unfolding transition of CLA was studied by performing DSC scans on CLA samples in the pH range 2 to 7. However, thermodynamic parameters could only be calculated in the pH range 7-4 (Table 6) since the thermal denaturation in this range was almost 90% reversible since transition peak was also observed upon down scan of the sample. The unfolding was irreversible in the pH range 3 to 2. At pH 2, the apparent T_m_ was found to be 327 K by two-state fitting. Hence, the irreversible nature of the thermal transitions prevented further analysis of the data. Fig. 10 shows a typical DSC thermogram of CLA corrected for buffer base line at pH 7 at a scan rate of 60 K h^−1^along with the fit of the transition data to a two-state model. The enthalpy (ΔH_v_) obtained as a two-state model fit at different pH (pH 7 to pH 4, dimeric range) scans, when plotted against the respective T_m_ yields a slope of 3.23±0.19 Kcal mol^−1^ K^−1^ (Fig. 11), is an estimate of the heat capacity (ΔC_p_). DSC scans were performed at different scan rates. Though the T_m_ values increase a bit with increase in scan rate (data not shown), the thermodynamic quantities ΔH_c_ and ΔH_v_ are independent of scan rate, hence the transitions are kinetically controlled and not thermodynamically controlled.

**Table 6:** Thermodynamic parameters for the thermal unfolding of 40 µM CLA by DSC at different pH values using a scan rate of 60 K h^−1^.

pH	T_m_ (K)	ΔH_C_ (kcal mol^−1^)	ΔH_v_ (kcal mol^−1^)	ΔH_C_/ΔH_v_
7.0	350.27±0.03	153.2±5.1	144.4±4.3	1.06
6.5	349.15±0.02	148.9±4.9	141.3±3.6	1.05
6.0	348.11±0.03	144.6±4.1	139.6±3.5	1.04
5.5	347.12±0.04	139.5±3.9	134.7±3.1	1.04
5.0	345.98±0.05	135.7±3.8	129.1±3.9	1.05
4.5	344.35±0.01	131.0±2.9	125.5±2.0	1.04
4.0	343.21±0.02	128.1±2.2	122.9±1.9	1.04
3.0	341.33±0.03	ND	ND	ND
2.0	326.97±0.02	ND	ND	ND

**Figure 12:**
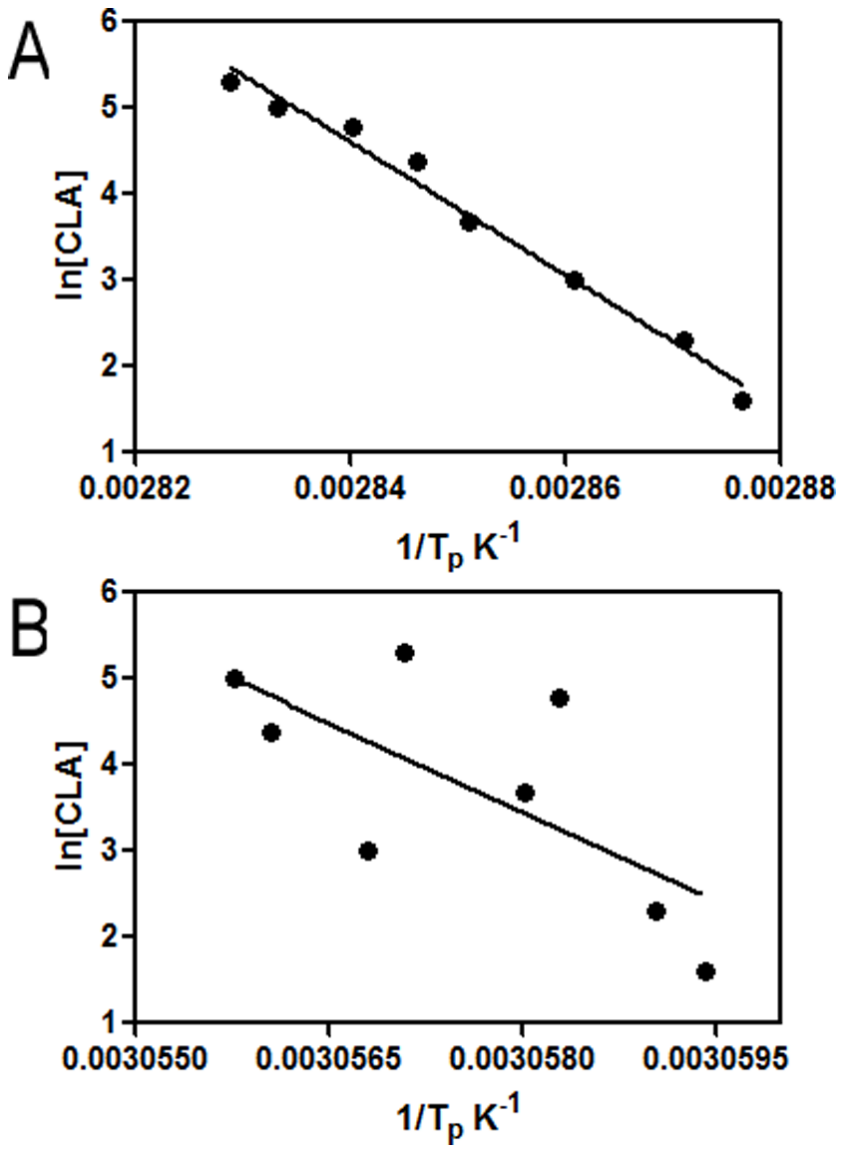
Dependence of T_p_ on protein concentration. Plot of ln[protein concentration] vs reciprocal of the transition temperature peak obtained from DSC scans at 60 K h^−1^. Protein concentration was used between 5 µM to 200 µM. T_p_ shifts towards higher temperatures as protein concentration increased at pH 7 (A) but T_p_ remains almost constant at pH 2 (B). The slope in the lower plot is smaller compared to that in the upper one, noting the 1/T_p_ values along the x-axis.

As pH decreased from 7 to 2, T_m_ also decreased indicating decreasing stability of the lectin. At pH 7, protein concentration had a significant effect on the T_p_ value, i.e., oligomeric protein undergoing dissociation could be related by Eq. 8. (Fig. 12) but the T_p_ value at pH 2 is independent of protein concentration, supporting a monomeric form.

## Discussion

It is difficult to purify large amounts of the turmeric lectin, as the yield is approximately 1 µg per gram of fresh root. The primary amino acid sequence and crystal structure of CLA are not available yet, so it is important to study CLA by biophysical methods under various conditions to characterize its assembly and stability. Disruption of the various non-covalent interactions that stabilize a protein structure, e.g. by thermal, acidic pH or chemical-induced denaturation, may result in partial or complete loss of structure and its biological activity, enriching our understanding of protein folding.

For most multimeric proteins, it is not possible to find conditions in which the folded monomer is stable with respect to the denatured state. This has given rise to the belief that inter-subunit contacts make a very large contribution to the stability of multimeric proteins, and that these interactions are important for subunit folding [31]. In the case of CLA, we are able to find a stable folded monomer at pH 2 (Figs. 2A, 2B). Hence in this case, inter-subunit interactions are not required for subunit folding. In multimeric systems, folding is typically coupled with subunit association [31]. Partially/completely folded, monomeric forms of a multimeric protein have been observed previously in a few cases. The dimeric CcdB (controller of cell division or death B protein) protein dissociates to monomer at pH 4, has similar secondary and tertiary structure, although dimeric interface is primarily hydrophobic [32]. The dimer of porcine mitochondrial malate dehydrogenase can be dissociated by relatively mild changes in pH. However, unlike in the present case, the monomeric form has different fluorescence properties. The monomeric form of the malate dehydrogenase enzyme present at pH 5 was shown by hydrogen exchange to be folded into a conformation similar to that adopted by the native dimer at pH 7 [33]. The dimeric enzyme 4-aminobutyrate aminotransferase also dissociates into monomers at pH 5 [34]. The monomers have similar secondary structure to the dimer. The *E. coli* enzyme glycan amide ribonucleotide transformylase (GART) also undergoes pH-dependent dimerization [35]. The soybean agglutinin that dissociates to monomeric form at pH 1.9 has similar secondary structure but shows different tertiary structure as well as fluorescence properties. The subunit association in soybean agglutinin is primarily due to ionic interactions rather than hydrophobic interactions at the interface [36]. In the present study we have been able to characterize the equilibrium stabilities of CLA, in both monomeric and dimeric forms. Far and near UV CD spectra, fluorescence emission maximum, showed no change in secondary or tertiary structure in the pH range 2 to 7 (Figs. 3A to 3E), while pH dependent DLS and size exclusion chromatography show dimer at pH range 7-4, going fully to monomers at pH 2 (Figs. 2A, 2B). As with the CcdB (controller of cell division or death B protein) protein [32], soybean agglutinin [36] and wheat germ lectin [37], the dissociation of the dimer into corresponding monomers occurs probably due to electrostatic repulsion among the individual subunits with the increase in positive charge in the system. A pH-dependent ANS study shows there was no binding of ANS in the presence of CLA dimer at pH 7 but ANS binding was observed for monomer at pH 2 (data not shown). The change in the charge distribution over surface of the protein might cause the hydrophobic patches to cluster at acidic pH; a similar result was observed for CcdB (controller of cell division or death B protein) protein [32]. Taken together with dynamic light scattering and gel-filtration studies, the spectroscopic observations suggest that, at pH 2, CLA exists as folded monomer with very similar secondary and tertiary structures. The CcdB (controller of cell division or death B protein) protein [32], Soybean lectin [36] and wheat germ lectin [37] show similar compactness in monomeric structure, whereas banana lectin [38], [39], lentil lectin [40] form molten globule without dissociating to monomeric form. Acrylamide quenching showing similar results at pH 2 and pH 7 (Fig. 6) also support a compact monomeric structure.

GdnCl-induced unfolding at pH 7 and pH 2 was also monitored by CD and fluorescence (Figs. 7A, 7B) and data analyzed by two-state models. The equilibrium unfolding of CLA in the presence of GdnCl was found to be a cooperative process in which protein molecule undergoes unfolding without formation of any partially unfolded intermediate at both pH values. By both CD and fluorescence, the C_m_ for GdnCl-induced unfolding was higher at pH 7 than at pH 2 (Table 3, Fig. 7). Similarly, ΔG° for GdnCl-induced unfolding was higher at pH 7 compared to that at pH 2 (Table 3, Fig. 7). These indicate that the native dimeric state (at pH 7) is more stable than the acid induced monomeric state (at pH 2). The C_m_ of the denaturant-induced transition was concentration-independent at pH 2, as expected for a monomeric state of the protein. Whereas, in the dimeric state C_m_ of the transition was highly concentration-dependent. One of the most important features of protein unfolding is the exposure of its hydrophobic core. This exposure is reflected in the positive change in the heat capacity of the protein during unfolding reaction. Thus, the change in heat capacity is directly proportional to the extent of unfolding and conversely the extent to which the apolar residues are buried in the native protein. The equilibrium thermodynamic parameters for the unfolding of dimer obtained from the fit of Eq. 7 are listed in Table 5. The small ΔC_p_ for unfolding of CLA reflects a buried hydrophobic core in the folded dimeric protein.

Temperature-dependent DLS showed transitions of hydrodynamic radius starting above 313 K, showing the monomer (at pH 2) is stable up to this temperature and the dimer (at pH 7) up to 333 K (Fig. 4), correlating with thermal unfolding studied at the same temperature region by the change in CD signal(Fig. 5).

The thermal stability of CLA was also characterized as a function of pH using DSC scans. At all the pH values listed in Table 6, the thermal unfolding transitions of CLA could be fit to a two-state model as N_2_↔2U at pH 7 to 4. The results presented in Fig. 2 and Table 6 reassert that CLA maintains its dimeric state over the pH range of 4–7. The correlation coefficient of 0.98 for the estimates of ΔC_p_ from ΔH_v_ versus T_m_ plots (Fig. 11) indicates that the ΔC_p_ values are pH-independent over the selected pH range. The value of ΔC_p_ obtained from this method is considered a better estimate [41] than that obtained directly as the difference between the native and unfolded baseline of a single DSC scan because of the errors associated with arbitrariness in determining the unfolded baselines [42]. According to equilibrium thermodynamics, any change in the oligomerization state of the protein during denaturation process should produce a concentration dependence of T_m_ [28], seen by plotting ln[protein] vs 1/T_p_ for pH 7 (Fig. 12A). The lack of concentration dependence (Fig. 12B) also proves the presence of monomeric form only at pH 2.

## Conclusions

The turmeric lectin unfolds via two-state pathway in both monomeric and dimeric states. The stabilization of the dimer derives primarily from interactions along the monomer-monomer contact interface of the CLA dimer. These inter-subunit interactions are not required for subunit folding. From available crystal structures of different lectins, the sugar binding ability appears to reside within a subunit. So it is important to maintain proper folding of each subunit to maintain biological function. Oligomerization stabilizes a protein in such a manner so that they can carry out their biological function efficiently. Though the turmeric lectin in our study forms monomer under acid-induced condition, it is still capable of sugar binding as its secondary and tertiary structures remain intact as in the dimer. This property may make CLA highly suitable for lectin-mediated drug delivery [4].
